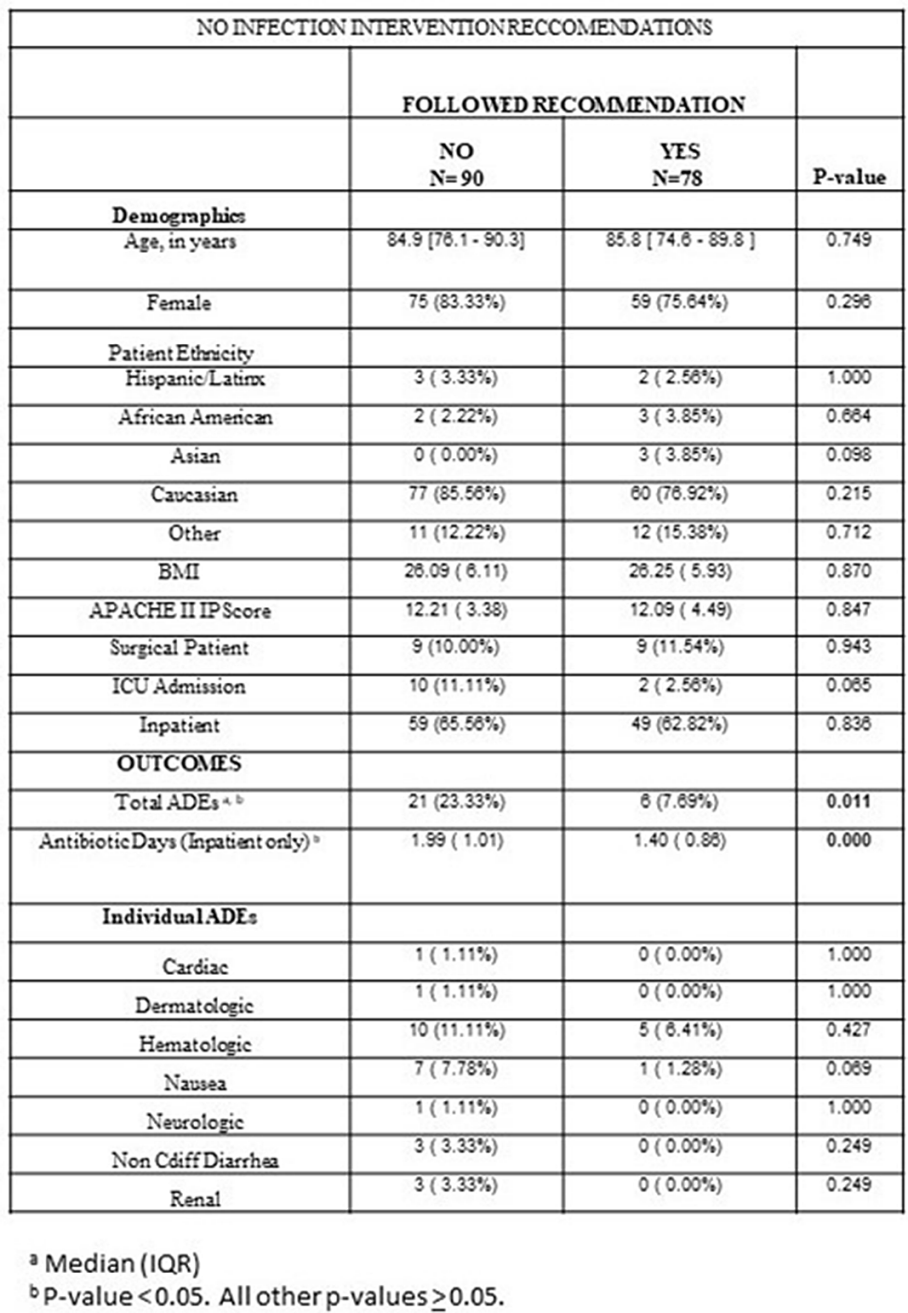# Adherence to Antibiotic Stewardship Program Associated with Shorter Course of Treatment and Fewer Adverse Events

**DOI:** 10.1017/ash.2021.55

**Published:** 2021-07-29

**Authors:** Patrick Mulligan, Nirav Shah, Mary Acree, Jennfer Grant, Urmila Ravichandran, Nader Ismail

## Abstract

**Group Name:** NorthShore University HealthSystem

**Background:** Prolonged antibiotic use has been attributed to an increased incidence of adverse drug events (ADEs). Cessation of unnecessary antibiotics would decrease length of treatment and may help prevent these adverse events. We evaluated whether an antibiotic stewardship intervention aimed at stopping unnecessary antibiotic usage would both shorten the duration of treatment and reduce ADEs. **Methods:** At NorthShore University HealthSystem, a 4-hospital, 832-bed system, we identified patients who were started on empiric antibiotics during a hospital admission between May 2, 2016, and June 30, 2018. Within 24 hours of antibiotic initiation, an infectious disease (ID) physician reviewed each patient chart. If the patient was unlikely to have a symptomatic bacterial infection, the ID physician left a note in the electronic medical record (EMR) recommending antibiotic cessation. Two physician reviewers retrospectively reviewed whether the treatment team accepted these recommendations and assessed potential ADEs for 30 days after the recommendation through inpatient and outpatient notes in the EMR. These ADEs were defined using previously published criteria. If the 2 reviewers disagreed on the presence of an ADE, an ID physician acted as the tie breaker. We compared the number of antibiotic days and the number of ADEs between cases in which the recommendations were followed and cases in which they were not. **Results:** We reviewed 168 cases: 78 (46.43%) followed recommendations and 90 (53.57%) did not. There were no significant differences in baseline patient characteristics between the 2 groups. There was a significant difference in total ADEs between the 2 groups: in 6 cases (7.69%) the recommendations were followed, and 21 (23.33%) they were not followed (*P* = .011). There was also a significant difference in antibiotic days between cases in which recommendations were followed (1.40 days) versus those in which they were not followed (1.99 days) (p < 0.001). **Conclusions:** Antibiotic-associated adverse events can cause harm to patients and increase healthcare costs, particularly when used for patients who are unlikely to have a bacterial infection. An antibiotic stewardship program to identify patients in an EMR who are unlikely to benefit from antibiotic use can decrease the length of total antibiotic usage and help prevent adverse events.

**Funding:** No

**Disclosures:** None

**Table 1.**